# ‘It was like an airbag, it cushioned the blow’: A multi-site qualitative study of bereaved parents’ experiences of using cooling facilities

**DOI:** 10.1177/02692163211059345

**Published:** 2022-01-20

**Authors:** Julia Hackett, Emily Heavey, Bryony Beresford

**Affiliations:** 1 Martin House Research Centre, University of York, York, UK; 2 Department of Behavioural and Social Sciences, University of Huddersfield, Huddersfield, UK

**Keywords:** Bereavement, grief, paediatric palliative care, cooling facilities, cold bedrooms, qualitative, cold cot, cooling blanket

## Abstract

**Background:**

Evidence on the benefits to parents of spending time with their child in the hours after their death means this is now routine practice. UK children’s hospices offer parents the opportunity to extend this period by using cooling facilities (i.e. cooled ‘bedrooms’; cooling blankets/mattresses) to slow deterioration.

**Aim:**

To explore parents’ experiences of using cooling facilities and beliefs about how it shaped experiences of the very early days of bereavement, and on-going grieving processes.

**Methods:**

Multi-site study involving in-depth interviews with parents bereaved in the previous 3 years. Grief theories informed data analysis, which employed narrative and thematic approaches. Eight hospices supported recruitment.

**Results:**

Twenty-two mothers and eight fathers were recruited, representing 25% of families approached. Duration of use of a cooling facility varied, as did the amount of time spent with the child. All parents treasured this period, valuing the way it eased separation from their child and gave some control over when this happened. They believed all bereaved parents should have the opportunity to use a cooling facility. Using a cooling facility supported parents’ engagement with grief tasks including acceptance of loss, processing emotional pain and facing changes to their lives brought about by their child’s death. Memories and mementoes created during this period served to support on-going connections with the child. Parents who used a cooling facility at a hospice reported benefits of the setting itself.

**Conclusions:**

As well as easing the very early days of loss, use of cooling facilities may influence longer-term bereavement outcomes.

**What is already known about the topic?**

- Newer grief theories argue that time with the body supports adaptive grieving. This practice is now widespread, though typically limited to a few hours followed by, if wished, visits to the funeral director.
- All UK children’s hospices have cooling facilities (e.g. cooled ‘bedroom’, cooling blankets/mattress). These delay deterioration and can extend the period before the child is transferred to a funeral directors by several days.
- The two existing and small studies report parents value highly the opportunity to use a cooling facility.

**What this paper adds?**

- There was considerable variation in how long parents used a cooling facility and the extent to which they spent time with the child during that period.
- Using a cooling facility meant the very early days of bereavement were less anguished for parents because their child remained in ‘the place of the living’ and they had greater control over when that ceased.
- Using a cooling facility supported engagement with the emotional and cognitive tasks of grieving, with the hospice setting and staff appearing to support this process.

**Implications for practice, theory or policy**

- Findings support the practice of *offering* bereaved parents the opportunity to use a cooling facility in the way currently provided by UK children’s hospices, though further research is needed to investigate whether place of use (hospice vs home) impacts experience and outcomes.
- In order to further support evidence-informed practice and parental decision-making, we need to understand extent of take-up, why parents some choose not to use a cooling facility, and impacts of cooling facility use on fathers’ and mothers’ bereavement outcomes in the longer term.

## Background

The death of a baby or child is a deeply profound and distressing experience.^1,2^ Compared to other bereaved people, parents are at greater risk of poor mental and physical health outcomes^2–4^ and complicated grief.^5^ Parents’ experiences of the period immediately after their child’s death may influence longer-term outcomes.^6–9^ During this time parents describe wanting to spend time with their child,^10–14^ and studies have reported the value and potential benefits of doing this.^9,13,15–18^ In response, this is now accepted as good practice in many countries.^19–21^ This marks a significant change. Previously, spending time with the child was strongly discouraged, with the belief that this supported breaking of ties,^22^ and ‘recovery’ from the loss.^23^ However, understandings of grief have shifted significantly in recent decades.^24^ Newer grief theories argue that relationships with the deceased continue, albeit in a different form. Thus rather than being something to avoid, providing opportunities to spend time with the body are seen as supporting the grief process, including acceptance of the reality of death.^25,26^

However, whilst many newly bereaved parents now have the opportunity to spend time with their child, it is typically limited to a few hours^18,27^ because of the need to cool the body to prevent deterioration. More unusual, and predominantly found in the UK, is the provision of ‘cooling facilities’ by children’s hospices to extend this period of time to a number of days. All have one or more dedicated rooms with specially designed air-conditioning systems (‘cold bedrooms’) and, when demand requires, they use portable air conditioning units and/or electrically cooled blanket /mattress in regular bedrooms. Families using such facilities have the option of staying at the hospice during this period. In addition, some hospices also offer parents the option of using a cooling blanket/mattress at home, though take-up of this appears to be low.^28^ Whilst practice varies, parents are typically offered use of such facilities for 5–7 days, after which the child’s body is transferred to a funeral director or their funeral. Their use is not restricted to children who die at the hospice and many hospices also accept referrals of families not previously known to them. Existing evidence indicates that, in at least some hospices, cooling facilities are used by the majority of parents.^28^

Despite their longstanding and widespread use in the UK, just two studies have investigated the parental experience. Both are single site studies, one (*n* = 16) collected free text data via a questionnaire.^18^ The other was a qualitative and concerned parents (*n* = 7) who experienced baby loss.^27^ Both report parents valued the opportunity to use a cooling facility as it allowed a more gradual relinquishment of the child’s body, and provided time to create mementoes and positive memories. Given that cooling facilities are used at a highly significant point in parents’ lives, with the potential for longer-term impacts on the grieving process, there is a clear case to extend this very limited evidence base.

This paper reports the second stage of a two-stage investigation of cooling facilities provided by UK children’s hospices. Stage 1 mapped provision and practices.^28^ Stage 2 was a multi-site qualitative study of parents’ experiences of, and their beliefs about, how using cooling facilities impacted the very early days of bereavement and on-going grieving processes.

## Methods

The study was qualitative, grounded in the phenomenological approach.^29^ Three bereaved parents acted as project advisors and contributed to refining recruitment processes, creating recruitment materials and developing and piloting of interview schedules. Leeds West (UK) NHS research ethics committee approved the study (REC reference: 18/YH/0487).

### Population

Eligibility criteria were that participants were: a bereaved mother or father (18+ years); whose child (aged 0–26 years) died at least 3 and no more than 24 months prior to recruitment; who had used a cooling facility provided by a UK children’s hospice; and that the hospice did not have concerns approaching the family regarding the study.

A children’s hospice is defined as a standalone service, which is nurse or doctor led, which is separate to hospital care and provides palliative and end-of-life care to children with life-limiting conditions and their families, with that care provided either in an in-patient/residential facility and/or in the family’s home.

### Sampling

Hospices participating in our Stage 1 survey^28^ were purposively selected as recruitment sites in order to represent: policy on permitted duration of use, use at home and acceptance referrals of families not previously known to the hospice. The target sample size was 30 parents, recruited from at least six hospices. Eight hospices supported recruitment. All eligible parents were invited to participate.

### Recruitment

The recruitment process was as follows. Hospices had the option to telephone families to seek permission to share brief study information. They then provided parents with brief study information and an expression of interest (EoI) form via post or face-to-face. Parents then returned the EoI form direct to the research team. The EoI form offered parents two options: either to receive a telephone call from a researcher (full study information was also posted); or to receive the full study information and a response form (requesting telephone call) via post. A reminder letter was sent after 2 weeks. A telephone call was then arranged with parents who were interested in participating, which provided further information and, if the parent wished to take part, arrangements for an interview were made.

The majority of study participants were recruited June–November 2019. We implemented a second, targeted wave of recruitment in November 2020 to increase representation of parents who used a cooling facility at home. Here, time since bereavement was extended to 36 months.

### Data collection

Parents could choose a face-to-face (in participants’ homes) or telephone interview, except where fieldwork took place during COVID restrictions (*n* = 3 interviews) where the options were telephone or video call. Where both parents wished to participate, individual or joint interviews were offered. Interviews were conducted by four researchers (the authors and AI (see Acknowledgements); all female, applied health service researchers, all have PhDs and previously unknown to participants; three are parents.)

Interviews comprised narrative^30,31^ and semi-structured components, with the former occupying the majority of the interview. First, parents were asked to tell their story of the time leading up to their child’s death through to their child’s funeral. Follow-up questions were used to supplement the told story where more detailed data were required with respect to: what parents did (cognitive, emotional, verbal, physical) when with and away from child; factors affecting the decision to use the cooling facility; longer-term impacts of using a cooling facility. A second set of questions explored experiences of specific hospice procedures and practices (reported elsewhere).

Interviews were audio-recorded and transcribed verbatim. Informed consent was obtained at the start of the interview.

### Data analysis

Our analysis was informed by three conceptual frameworks. First, we understood the experience of loss as something which abruptly places an individual in a liminal, or in-between, state which can be deeply uncomfortable and disorientating, requiring emotional and cognitive work to move from this state to successfully adapting to the loss.^32^ Second, we drew on notions of bereavement as the loss of someone to the ‘place of the dead’ (e.g. funeral directors, graveyard).^33–35^ Finally, we used Worden’s^26,36^ task-based model of grief which sets out the emotional and cognitive tasks which support positive adaptation to loss.

Prior to working with the transcripts, researchers leading on the analysis listened to the audio-recordings of all the interviews. Parents’ stories were analysed using structural and thematic analytic approaches.^31^ A structural analysis created an extended, chronological commentary of each parent(s) story. Commentaries included detailed summaries (including verbatim extracts) of significant moments and events, including parents’ accounts of their thoughts and feelings and current reflections on those times. Each commentary also included a record of researcher reflections and hypotheses. A thematic analysis was used to investigate parents’ beliefs about the impacts of the opportunity to use a cooling facility on the early days of grief and longer-term adaptation, and the role of cooling facilities in supporting engagement with grief tasks^26^ and factors affecting parents’ engagement with those tasks. Here relevant data in transcripts was indexed and then summarised. ‘Analytical notes’ were drafted which, informed by the conceptual frameworks referred to above, collated, described and compared parents’ accounts and recorded researcher observations and reflections. These notes were developed and refined iteratively through a cycle of writing, critical review and discussion within the team.

## Results

### Sample

Ninety-six families received study information. Twenty-two mothers and eight fathers were recruited, representing 23 families. Seven interviews were joint interviews with a mother and father (in two, the father did not stay for the whole interview). The remainder were individual interviews (mothers: *n* = 15; fathers: *n* = 1). Mean interview length was 92 min (range: 39–179 min). Parents had been bereaved between 5 and 35 months (median = 13 months). Table 1 provides an overview of other sample characteristics.

**Table 1.** Additional characteristics of sample.

Age of child when died	
6 weeks or less	6
7 weeks–12 months	2
1–4 years	5
5–10 years	4
11–18 years	5
19+ years	1
Place of death	
Hospital	9
Hospice	9
Home	5
Other	1
Place of cooling facility use	
Hospice	20
Home	3
Previous users of the hospice	
Yes	8
No	15
Other siblings at time of child’s death	
Yes	14
No	9
Parents’ ethnicity (*n* = 30)	
White British	20
Other^a^	10
Destination after use of cooling facility	
Funeral directors	17
Funeral	6

a Aggregated due to small numbers. Included: Black or Black British; Asian or Asian British; White (non-British).

### How cooling facilities were used

The majority of parents had used cooling facilities at the hospice (*n* = 20/23 children represented), see Table 1. Duration of use ranged from 1 to 14 days with most using for 5 to 7 days. When used at a hospice, the majority of families (*n* = 15/20) were also resident, though two did not stay for the entire period. Five families chose not to stay at the hospice. Typically, parents’ described a decrease over time in the frequency and length of time spent with their child. Two families chose not to visit their child (one co-resident in the hospice, one stayed at home).

Parents spent times with their child in different ways. All described sitting with, looking at and talking to their child, and doing things with them (e.g. reading, playing music, taking on walks, painting nails). Many reported touching or stroking (typically the child’s hair) or, less frequently, holding or lying with the child. Memento making also took place (photographs, cutting locks of hair, hand/foot casts/prints). A few engaged in physical care of the body (e.g. washing). However, typically, this was handed over to staff. Finally, parents described very brief visits, often presented as ‘checking on’ the child, or to say good morning/night.

### Overall descriptions of using cooling facilities and its perceived impacts

All parents described the time using a cooling facility as being highly significant and of great value.

Utterly precious. . .such an important starting point (4218, mother)

The opportunity to just spend the time I did have with my child has been immense. (0508, mother)

None regretted using a cooling facility. In recalling this time, many parents noted the juxtaposition of positive experiences within a time of great anguish.

In a very emotional time it was as good as it could have been really. It’s the worst time of your life. It’s making the worst time of your life as good as it could be. (2221, father)

They also believed using a cooling facility had affected grieving processes and outcomes, both in the short and longer term.

I think we’d have just been a bit more, a bit more down really, cos obviously that experience just enlightened everything and just made everything a little bit more positive and if that wasn’t there, I don’t think we’d have coped as well. (2213, mother)

Importantly, there was strong consensus that all bereaved parents should have the opportunity to use a cooling facility. In the remainder of this paper, we report findings on why parents consistently regarded the experience of using cooling facilities so positively, and why their use might impact immediate and longer-term experiences and effects of bereavement.

### Impacts of using cooling facilities on experiences of loss and separation

A key theme within parents’ accounts was the notion that cooling facilities meant their child remained, for a while, in the ‘place of the living’, and the moment of separation to the ‘place of the dead’^33–35^ was, to some degree, under their control. As a result, parents believed the early days of grief were less distressing and disorientating.^37^

*Mother*: It just was a kind of gentle way to deal with the most shocking mind-blowing thing I’ve ever known. . .and I think that was a real kind of healing process and just coming to terms with the reality of what had happened.

*Father*: I think it created a buffer between life with [name of child] and life without. (1006, mother and father)

I think it would be hard just to kinda completely break it off and be like, OK, that’s it, you can’t see them anymore. So I think it definitely gave us like that little bit of extra time. . .you haven’t really got your head around it, but it’s not that abrupt. (0512, mother)

It’s acclimatising to that change which is much more precious and much more valuable and much more real if you can do it without him just disappearing off to somewhere with strangers who I don’t know. (4218, mother)

A number of different factors contributed to this. First, the fact that the child was not in a mortuary or funeral directors was, in itself, deeply comforting.

When they said, you know, they’re gonna remove the tube, for me it was like and what then? I just couldn’t imagine she will go to a cold, empty fridge and be there on her own. (4223, mother)

I didn’t want him on a slab. I didn’t want that for him, he was a baby, he needed to be cuddled. (3402, mother)

Second, the child remaining in ‘the place of the living’ meant some things about the parent-child relationship, and the things parent did with their child, remained unchanged and withdrawal from these things could be gradual. For parents who had experienced baby loss, it allowed the creation of memories with their baby in ‘the place of the living’. Being able to do these things was presented as a salve to parents’ emotional distress, making the actual transfer to the ‘place of the dead’ more bearable.

It helped me considerably because I was able to go in and just be. . .to hold him, to be near to him, to speak to him, to caress him, to look at him. Because it just allowed us to let go, to say goodbye. (2916, mother)

It gave us time, knowing he’s gone but you’ve still got longer, which was everything. (3402, father)

Finally, parents felt relatively in control of when their child transferred to the ‘place of the dead’. As a result, the move to the funeral directors or funeral almost always happened when the parent felt ‘ready’, and the thought of this happening was no longer intolerable. (We note, however, that for a very small minority, the timing of the move was dictated by the needs or wishes of other family members, or by religious requirements.)

[*It*] just felt like, you know, we’ve had a nice time with him now and, and this is the next stage. (4224, mother)

The time at the hospice prepared me mentally to say goodbye and I was ready (4222)

For the majority, no further time was spent with the child: visits to the funeral director either did not take place or only happened once or twice.

### The role of cooling facilities in supporting grief work

Worden’s^26,36^ task-based grief theory argues that successful adaptation to bereavement requires engaging with four tasks of grief, each of which may be returned to on multiple occasions. In this section we report findings on how using cooling facilities may support engagement with each of these tasks.

#### Accepting the reality of loss

A sense of unreality and disbelief are almost universal reactions to a significant loss.^1,2^ Acceptance of the reality of loss is foundational to adaptive grieving and positive bereavement outcomes. A strong and consistent theme in parents’ accounts was their conviction that using a cooling facility supported this process. First was the physical evidence of their child’s body, both visual appearance and being cold to the touch. Being able to freely and repeatedly return to ‘check’ this evidence was important.

I think there is something about being around a dead body that is kind of healing. I think it gives you a real congruence that person is gone, they’re not there, they haven’t just vanished in a sanitary puff of smoke: this is what death looks like, and at this point it’s sort of peaceful (1006, mother)

A further aspect of the physical evidence of death was deterioration of the body. Some explicitly referred to this as helpful, it was not consistently regarded as important to acceptance. Indeed some parents avoided seeing deterioration.

*Mother*: And in some ways I do think I needed to see that, I needed to know he was really gone, and this is what happens when someone dies, and I think I needed to have a sense that it’s not like he’s out there somewhere and I should really keep trying, I think it gave me that sense of. . .

*Father*: . . .closure. (1006, mother and father)

Critically, parents believed assimilating the knowledge of their child’s death, and a psychological acceptance of it, was made easier by the fact they still ‘had’ their child.

I think maybe makes it more real. I can’t deny that he is dead because I lived with his body for four days after it had happened. And he wasn’t ripped away from me, I think is quite important. I think that might have been the case if we hadn’t had those four days it would have felt like he was ripped away and we weren’t in control. We had him home for four days and then [*name of father*] carried him out of the house. (4224, mother)

#### Processing the pain of grief

Facing and dealing with, rather than suppressing, emotional pain is Worden’s second task of mourning. Parents identified a number of consequences of using a cooling facility that supported the very early days of engaging with this task. First, whilst no less profound, parents believed the initial shock and pain of loss was ‘softened’ because it did not happen in a single traumatic moment.

It was like an airbag, it cushioned the blow. (2611, mother)

Some specifically identified that this left them feeling more able to begin to face their feelings. In addition, going forward, memories of this period were an on-going source of comfort, soothing feelings of loss and pain.

Both at the time and longer term. . .we have something positive to reflect upon following something so harsh. . . .It might sound strange but making memories with [*child*] [*is*] something which [*is*], a point of reference that you can go back to. (2916, mother)

Parents who used cooling facilities at a hospice described the value of being in a setting where death and grief were ‘normal’, and talking about them was not avoided. This facilitated expressing emotions.

You’re [*in*] a place and a structure that actually does talk about it [*death*]: this is what we do here, it is normal, your experience is absolutely normal, even though it’s unique, and we can deal with it. I think that was the biggest thing is knowing that they can cope with. . .the event of death. So it’s OK to speak about how you’re feeling. (4218, mother)

Many also believed being away from usual routines and the home environment had ‘made’ them have conversations about their thoughts and feelings. Parents with other children, particularly younger ones, said being able to hand over care of siblings was key to them being able to engage with processing their feelings.

I think it was just the fact that everything [siblings’ *needs*] was being met and the pressure was off me. . .And [*things* siblings’ *workers did*] that really perked them up and that was good for us to see. (1003, mother)

Less clear was whether the child’s on-going presence also enabled facing and engaging with their emotions, though certainly some parents did make this connection.

I don’t think it [*using at cooling facility at home*] would have been good for any of us [*mother, father and surviving teenage/young adult children*] because I don’t think anybody would have talked. So when we were together and there were no Wi-Fi we actually talked to each other about things. So if that facility weren’t there and we’d had to have gone straight back home I don’t think any of us would have coped that well, to be honest. . .But to just spend that time together and still laugh and cry with her close by, I feel made, you know, the whole difference. (0509, mother)

Finally, some parents noted the advice and practical support of hospice staff, where they wished, with funeral planning and legal processes saved them from being distracted by these tasks and allowed space for reflection.

#### Adjusting to a world without the dead person

Bereavement can bring multiple and wide-ranging changes to a person’s life. The process of adjustment to these changes is long-term, with new realities emerging or needing to be faced over time. There was evidence in many parents’ accounts of very early work engaging with this task. This appeared to be supported by the comfort gained from their child’s on-going presence. Engaging with this task was more noticeable where parents had chosen to use a cooling facility at a hospice and for a greater number of days.

For many parents, a key area of adjustment, and abruptly evident to them from the outset, was that daily life was no longer occupied by the demands and routines associated with their child’s care. A number noted that first experiencing this change away from the home environment had been helpful. It had meant they returned home having had time to prepare for this, sometimes with new patterns to their day already established.

It was a nice transition and it helped me adapt to the new pace of my life where my days are so much longer because they’re not filled with caring for him. It did help because it was a big transition. It was a nice adjustment to the new slower pace of my life and I think that helped me psychologically, it helped me adjust. (2611, mother)

. . .So being somewhere different with a different routine. . .that really helped to break that [*routine*]. So that by the time we got home, being in a different pattern to what we had been felt normal. . .I think that probably was really important. (1006, mother and father)

Many parents, at the suggestion and encouragement of staff, left the hospice premises during their stay. Often what they did centred on ‘doing things for the first time’ since the child’s death (e.g. being at home without the child, going to a familiar place without the child). Returning to their child’s body, and the support and seclusion of the hospice, helped them undertake and cope with these activities.

*Father*: Yeah, it was really odd feeling that you could just leave the hospice and not worry; it was the first time we’d been able to leave her without worrying and they were. . .

*Mother*: Yes

*Father*: . . .they were really trying to encourage you to go out and try and do something and just I think, a slower step into the process of leaving her and not having her (4225, mother and father)

#### Finding enduring connections with the dead person in the midst of embarking on a new life

This grief task concerns finding ways to carry on with life whilst simultaneously remembering and commemorating the dead person. Parents consistently identified the opportunity to create new and positive memories of their child as a key benefit of using the cooling facility. Where a child had been dependent on medical technologies, parents now had memories of their child without being attached to such equipment. For others it meant they had memories of their child being peaceful, or that memories of a traumatic hospital admission had been overlaid.

We have something positive to reflect upon following something so harsh. Spending time with him. . .making memories with [*child’s name*]. . .a point of reference that you can go back to. (2916, mother)

Where a baby had never been discharged home from hospital, memories of being with them, and doing things they had imagined and looked forward to, were very precious.

All I remember really is just sitting with him and holding him. I think it just gave us that bit of time that we didn’t have; I was able to take him out for a little walk as well in the little forest outside. It was nice to do that, cos I obviously hadn’t got to take him outside before. (2604, mother)

The opportunity to create mementoes (i.e. physical reminders) was something that all parents valued highly. All had taken photographs and made physical ‘imprints’ (e.g. finger/hand/foot prints/casts, locks of hair). Parents varied in whether they actively participated in creating these. Sometimes the state of the body meant hospice staff did this, or parents simply preferred not to be involved. Regardless, these mementoes represented a treasured, enduring physical link to the child.

It’s nice little things that you can take forward with you, it’s like longer lasting. (2915, mother)

## Discussion

### Main findings

Parents varied in the duration and way they used the extended period of time their child remained ‘with’ them. However, they were unanimous about value and benefits of using a cooling facility, and that all bereaved parents should have this opportunity.

Parents believed it had softened their immediate anguish and made the final separation from the child more tolerable. In addition, using a cooling facility supported parents to begin to engage with grief, or mourning, tasks.^26,38^ Thus as well as easing the immediate experience – which in itself may position parents to be better able to cope with their grief – use of cooling facilities has the potential to impact bereavement outcomes. A number of different mechanisms or processes appeared to be at play. Seeing the child’s body, often on multiple occasions, helped acceptance of death. The child’s on-going presence lessened the disorientation and distress typically experienced.^32^ This supported acceptance and meant parents felt able to face, rather than suppress, painful emotions. Those using a cooling facility at a hospice found being away from usual spaces and routines (which could be used to retreat from other family members or avoid facing distressing emotions), supported helpful times of conversation and reflection. Furthermore, in this setting, death was ‘normal’: there was no avoidance of, or discouragement to display, emotions. Parents also believed the child’s on-going presence meant contemplating life without them, and the often very significant changes that would bring to daily life and routines, had been less painful and meant they were better prepared for these changes. Parents who used a cooling facility at a hospice felt doing that early work on mentally adjusting to a different life away from the home environment had been helpful. Some found the rhythms of hospice life had helped regularise daily routines significantly disrupted by the child’s care needs and hospital admissions. Empathetic encouragement by staff to begin re-engaging with their local communities and everyday situations was valued. Finally, using cooling facilities allowed parents’ final memories of their child to be positive. This, together with the physical mementoes created, provided lasting and comforting connections with their child.

### What this study adds

Findings align with previous studies^18,27^ but also significantly extend and develop the evidence base. This is because we investigated both the experience of using cooling facilities *and* their potential impact on grieving and longer term outcomes. Furthermore, we grounded this analysis and interpretation in existing theoretical and conceptual frameworks.^26,32,36,38^ The size and characteristics of our sample mean our findings have wider applicability than previous studies.

The notion that cooling facilities delay the time a child is transferred to ‘the place of the dead’^33–35^ brought important new insights. It highlighted that cooling facilities do much more than simply allow parents more time *with* their child. This is clearly an insufficient understanding given not all parents visited their child, and the pattern and duration of visits was highly variable. Rather, it is the delaying of the moment of transition from the ‘place of the living’ to the ‘place of the dead’, which is, in itself, of great solace: both at the time and in the months and years ahead. Furthermore, when this transition happened it was less painful because parents had some control over its timing.

These findings carry important implications for the way cooling facilities are provided. They indicate that the practice of using cooling equipment to extend ‘visiting times’ to children already placed in mortuaries or funeral directors cannot offer the same benefits. They also question the appropriateness of arbitrary limits on duration of use. Finally, they suggest that facilities for children within funeral directors and mortuary services should be designed to more closely resemble ‘the place of the living’ and visiting policies adjusted to allow more spontaneous access.

We also found that using cooling facilities supports engagement in grief tasks^26^: work necessary to successful adaption to loss and reduced risks of poor bereavement outcomes. Importantly, it was not just the child’s continued presence that supported engagement in such tasks. Where parents used cooling facilities at a hospice, being in a setting where death was normalised and staff were empathetic and highly experienced supported engagement in grief work. Taken together, these findings make the case for research that systematically evaluates the impacts of cooling facilities on parents’ grieving processes and bereavement outcomes. They also offer an initial theory^39,40^ to inform the evaluation design.

Furthermore, though restricted in our conclusions by the small number in our sample who used cooling facilities at home, our findings raise questions about support provided to families who choose to use a cooling facility at home. In contrast to when in-house cooling facilities are used, UK hospices’ approaches to supporting families at home are much lighter touch, often focussing on care of the body.^28^ Given that hospitals (particularly maternity services) are increasingly offering cooling facilities for use at home, it is important that future research considers both hospices and hospitals as providers.

Finally, our findings offer support to the argument that all bereaved parents should be offered the opportunity to use a cooling facility (in ways similar to those represented in this study). In the UK, the location of hospices, and their capacity, currently make this unfeasible and places the financial burden exclusively on the charitable sector. This points to the need for debate on the role and contribution of statutory services in bereavement support. Evidence of the psychological, physical, social and economic costs of bereavement^2–5^ strengthens the case for this. Beyond the UK, preliminary evidence on provision of cooling facilities by children’s hospices^28^ suggests this is highly unusual. Here the question is more fundamental: should non-UK hospices look to routinely offer such facilities?

### Strengths and limitations

The sample fulfilled the sampling framework. However, less than a quarter of families approached were recruited, and the majority of the sample were mothers. This is a lower response rate than other studies of bereaved parents.^41^ However, this is the first study specifically focussing on the highly traumatic period immediately after death and, compared to some studies, parents were relatively recently bereaved. Both these factors are likely to have affected parents feeling able to participate. Overcoming these difficulties will be continue to be challenge and researchers will need to work in partnership with bereaved parents to develop recruitment strategies. In addition, few families had used a cooling facility at home, though we note the sampling pool is very small.^28^

These limitations constrain the conclusions which can be drawn. To further test our conclusions, research comparing parents who used and did not use cooling facilities is needed. Finally, whilst a range of ethnic, religious and cultural backgrounds were represented in the study, our sample size was too small to explore their possible impacts on experiences of using cooling facilities. We know very little generally about how these factors impact parental bereavement experiences^19,42^ and is clearly something which should be prioritised in future research.

## Conclusion

Parents differed in how long they used cooling facilities and how they used that time. However, all believed it had positively impacted on their experiences of the early days of bereavement and grieving processes. For the UK, the findings raise implications for current models of provision. In other countries, where use of cooling facilities is much more unusual, it raises the more fundamental question of whether they should be routinely integrated into paediatric palliative care provision.
